# Anti-Inflammatory and Antioxidative Stress Effects of Oryzanol in Glaucomatous Rabbits

**DOI:** 10.1155/2017/1468716

**Published:** 2017-01-12

**Authors:** Shital S. Panchal, Rajesh K. Patidar, Abhishek B. Jha, Ahmed A. Allam, Jamaan Ajarem, Shital B. Butani

**Affiliations:** ^1^Institute of Pharmacy, Nirma University, Sarkhej-Gandhinagar Highway, Ahmedabad 382481, India; ^2^Department of Zoology, College of Science, King Saud University, Riyadh 11451, Saudi Arabia; ^3^Department of Zoology, Faculty of Science, Beni-Suef University, Beni-Suef 62511, Egypt; ^4^Nahda University in Beni Suef, Beni-Suef 62511, Egypt

## Abstract

*Purpose*. *γ*-Oryzanol works by anti-inflammatory and radical scavenging activity as a neuroprotective, anticancer, antiulcer, and immunosuppressive agent. The present study was conducted to investigate effect of oryzanol in acute and chronic experimental glaucoma in rabbits.* Methods*. Effect of oryzanol was evaluated in 5% dextrose induced acute model of ocular hypertension in rabbit eye. Chronic model of glaucoma was induced with subconjunctival injection of 5% of 0.3 ml phenol. Treatment with oryzanol was given for next two weeks after induction of glaucoma. From anterior chamber of rabbit eye aqueous humor was collected to assess various oxidative stress parameters like malondialdehyde, superoxide dismutase, glutathione peroxidase, catalase, nitric oxide, and inflammatory parameters like TNF-*α* and IL-6. Structural damage in eye was examined by histopathological studies.* Results*. In acute model of ocular hypertension oryzanol did not alter raised intraocular pressure. In chronic model of glaucoma oryzanol exhibited significant reduction in oxidative stress followed by reduction in intraocular pressure. Oryzanol treatment reduced level of TNF-*α* and IL-6. Histopathological studies revealed decreased structural damage of trabecular meshwork, lamina cribrosa, and retina with oryzanol treatment.* Conclusions*. Oryzanol showed protective effect against glaucoma by its antioxidative stress and anti-inflammatory property. Treatment with oryzanol can reduce optic nerve damage.

## 1. Introduction

Oxidative stress is the major contributing factor for cellular and metabolic deregulation. It is persuaded by reactive oxidizing agents and free radicals. In recent years, naturally occurring phytochemicals with antioxidant property have been an emerging interest because of their expedient effect on human health as they provide protection against oxidative degradation which is responsible for cell dysfunctions and neurodegeneration. Lessening of oxidative stress due to free radicals, exclusively at mitochondria level, appears to be defensive. Antioxidants, that is, gingko, ubiquinone, and melatonin, polyphenolic flavonoids in green tea, coffee, or red wine, and anthocyanosides existing in bilberries, have been proved to have therapeutically beneficial effect in such context [1].

Glaucoma is stated as extremely elevated intraocular pressure (IOP) and progressive degeneration of the optic nerve, which is characterized by selective apoptosis of retinal ganglion cells (RGCs) and optic nerve head structural modulation [2]. It is the major cause of blindness after cataract worldwide. According to WHO report 2012, around 285 million people are estimated to be visually impaired worldwide. About 90% of visually challenged population reside in developing countries and 82% of population existing with blindness are at and above 50 years [3]. Numerous mechanisms that have been concerned for RGCs death in glaucoma include deprivation of neurotrophic factor, ischemia or hypoperfusion, activation of glial cells, glutamate offered excitotoxicity, and unusual immune reactions [4]. In glaucoma RGC apoptosis follows oxidative stress, ROS generation, ATP deprivation, and cytochrome C release. No matter how the axon degenerates, RGC's loss follows apoptotic pathway and neurotrophin deficiency due to suspended axoplasmic transport. Further axonal collapse occurs because of native astroglial cells participation and resides in head of the optic nerve and lamina cribrosa. Hence axons suffer extensive damage and follow Wallerian degeneration with severe microfilament and microtubule breakdown as well as mitochondrial swelling [5]. Existing therapies are aimed at managing the illness but not curing or reducing the advancement of glaucoma. The U.S. Food and Drug Administration has approved five categories of drugs that temporarily improve the symptoms, that is, alpha-agonist, beta-blockers, carbonic anhydrase inhibitors, parasympathomimetic agents, and prostaglandin analogs. Oryzanol (OZ) is a physiologically active compound present in* Oryza sativa* (Rice) bran oil (RBO) at level of 1-2%. It is a mixture of 4-hydroxy-3-methoxy cinnamic acid (ferulic acid) esters like cycloartenyl ferulate, 24-methylene cycloartanol ferulate, *β*-sitosterol ferulate, and campesteryl ferulate [6]. OZ has been stated to possess comprehensive assortment of biological actions; in specific it retains antioxidant/free radical scavenging activity [7]. In experimental rats OZ has been reported for anticarcinogenic and antihyperlipidemic activity [6]. Anti-inflammatory activity possessed by OZ is by virtue of its NF-kB inhibitory nature, so it can inhibit activation and nuclear translocation of nuclear factor kappa-B (NP-kB) in macrophages, which predominantly plays an important role in exacerbation of inflammatory reactions in neurons followed by damage of optic nerve and RGCs loss [8, 9]. Recently an investigation conducted on SH-SY5Y cells by oryzanol-rich fraction against H_2_O_2_ induced neurotoxicity has revealed its neuroprotective activity [10]. Ferulic acid downregulates ICAM-1 mRNA expression, level of microglia, and macrophages and simultaneously reduces the inflammation induced oxidative stress and consequential apoptosis due to cerebral ischemia and reperfusion injury in rats [11]. Ulcerative colitis murine model investigation has stated nitric oxide (NO), TNF-*α*, and IL-1*β* attenuation aspects of OZ [12]. Keeping this in mind the study has been targeted towards implementation of OZ treatment in glaucoma.

## 2. Methods

### 2.1. Chemicals and Reagents

All bioanalytical assay kits were acquired from Lab care diagnostics Pvt. Ltd. India. The oryzanol was purchased from Tokyo chemical industry Co. Ltd., Tokyo, Japan. Pilocarpine was procured from FDC limited, India. Xylocaine was obtained from Astra Zeneca Pharma India limited. Phenol and almond oil were procured from central drug house, India. TNF-*α* and IL-6 assay kits were purchased from B D Biosciences, USA.

### 2.2. Animals

The experimental protocol involving animals were approved by the Institutional Animal Ethics Committee (IAEC) of Institute of Pharmacy, Nirma University. (IP/PCOL/MPH13-1/006). Adult New Zealand White Rabbits of either sex weighing 2-3 kg were selected for the study. The animals were familiarized to the laboratory surroundings for 2-week period. They were sustained at 25 ± 4°C and 12/12 h of light-dark cycle and were given standard rabbit feed (Pranav Agro Industries Ltd., Pune, India) and water ad libitum.

### 2.3. Acute Glaucoma Model

Animals were randomly divided into normal control (*n* = 6), acute control (*n* = 6), OZ treated (*n* = 6), and pilocarpine treated (*n* = 6) groups. Acute model of glaucoma was developed by earlier described procedure [13]. Before induction of acute glaucoma, the basal IOP was measured by Schiotz Indentation tonometer in all animals after instillation of the local anesthetic Xylocaine 2% eye drops. This process was repeated each time before the measurement of IOP. After IOP recording, 1% CMC was administered in normal control and acute control animals. Two to three drops 1% OZ solution prepared in 1% CMC was instilled topically into the eye for treatment as a single dose. 1% pilocarpine was used as standard treatment in animals. It was applied topically as a single dose of two to three drops. After 15 minutes of vehicle and drug administration, intravenous 5% dextrose solution (15 ml/kg) was infused through marginal ear vein to induce ocular hypertension. The intraocular pressure (IOP) changes were noted by tonometer at 15, 30, 45, 60, 90, 120, 180, 240, 300, and 360 minutes (till normal pressure is achieved). The tonometer used for IOP measurement was calibrated by open manometric calibration.

### 2.4. Chronic Glaucoma Model

Animals were randomly divided in to normal control (*n* = 6), chronic control (*n* = 6), OZ treated (*n* = 6), and pilocarpine treated (*n* = 6) groups. Chronic model of glaucoma was developed by formally described procedure [14]. 150 *μ*L phenol, prepared in almond oil (5%), was injected in subconjunctival region in the four quadrants of rabbit eyes to produce scarring in the aqueous humor (AH) pathway-causing increase in IOP without apparent macroscopic or microscopic damage to the eye. The injections were repeated (once a week) for next two weeks till IOP was elevated completely. IOP was recorded every day by Schiotz Indentation tonometer during induction period. After 3 weeks of induction period 1% CMC was administered in normal control and disease control group while 1% OZ solution (prepared in 1% CMC) and 1% pilocarpine were instilled topically as test and standard in their respective groups for 2 weeks once a day, two to three drops.

#### 2.4.1. Aqueous Humor Collection

Aqueous humor (AH) samples were collected by method described by Gupta et al. [15]. In brief, AH was collected carefully from the anterior chamber of rabbit's eye using 27-gauge needle after administration of diazepam 1 mg/kg i.v. and ketamine 25 mg/kg i.v. without causing any injury to the iris or lens throughout the procedure. After collection, samples were immediately stored at temperature (−20°C) till the performance of various biochemical analysis from AH.

#### 2.4.2. Estimation of Total Protein

Total protein was estimated in AH as per Lowry method [16]. Briefly, AH (10 *μ*L) was allowed to be diluted using 1 N NaOH (990 *μ*L) and kept for reaction with copper reagent (4 mL). 10 min later, Folin's reagent (0.5 mL) was added and mixed using vortex mixture. Next, the samples were placed in dark at room temperature for 30 min. At 620 nm absorbance was estimated using spectrophotometer. Bovine serum albumin (BSA, sigma chemicals, USA) was used as standard.

#### 2.4.3. Estimation of Antioxidant Parameters

Malondialdehyde (MDA) levels were measured as per the method described by Ohkawa et al. In brief, 0.1 ml tissue homogenate was mixed with 0.2 ml 8% sodium dodecyl sulphate. Resultant solution was mixed with 1.5 ml 20% glacial acetic acid and 1.5 ml 0.8% thiobarbituric acid; thereafter pH 3.5 was adjusted with NaOH. Mixture was diluted up to 4 ml with distilled water. Solution was heated at 95°C for 60 min and then cooled by running tap water. Next to this 5 ml of 15 : 1 v/v n-butanol and pyridine was added. The mixture was mixed thoroughly and shaken rapidly. Mixture was centrifuged for 10 min at 4000 RPM and the supernatant was collected. Its absorbance was measured at 532 nm [17].

Nitrite (NO^−2^) levels were measured by method reported by Tracey et al. The method utilizes Griess reagent. In brief, 6 *μ*L of plasma sample, 44 *μ*L dH_2_O, 20 *μ*L 0.31 M pH 7.5 phosphate buffer, 10 *μ*L 0.86 mM NADPH, 10 *μ*L 0.11 mM FAD, and 1.0 U/ml nitrate reductase were mixed together in well plates. Resultant mixture was incubated for 1 hr at room temperature. Next, 200 *μ*L Griess reagent was added to each well and allowed to incubate at room temperature for 10 min. Then absorbance was measured at 549 nm [18].

Glutathione peroxidase (GPx) level was measured by method described by Mohandas et al. In brief to the reaction mixture composed of 0.05 M pH 7.0 phosphate buffer, 1 mM EDTA, 1 mM sodium azide, 1 U glutathione reductase, and 1 mM GSH, 0.2 mM NADPH 100 *μ*L of the tissue homogenate equivalent to 6 mg protein/ml was added. The final mixture was treated with 0.25 mM hydrogen peroxide and the enzyme activity was initiated at 37°C. The absorbance was measured at 340 nm [19].

Superoxide dismutase (SOD) was estimated by the method suggested by Kovačeva et al. In brief, 750 *μ*L tissue homogenate was assorted with 600 *μ*L ice-cold ethanol-chloroform mixture mixed at 5 : 3 v/v. Resultant solution was thoroughly mixed and then centrifuged for 45 min at 4°C. Supernatant was diluted with 50 mM pH 7.8 phosphate buffer, 5 mM EDTA, 40 mg/L bovine serum albumin, 0.2 mg/ml nitroblue tetrazolium, and 0.1 mM xanthine oxidase and xanthine. The absorbance was taken at 546 nm for 10 min [20].

Catalase (CAT) levels were measured using method described by Hugo. To 2 ml tissue homogenate 1 ml 30 mM H_2_O_2_, prepared in 50 mM pH 7 phosphate buffer, was added. Reduction in absorbance was observed at 240 nM [21].

#### 2.4.4. Estimation of Inflammatory Mediators

TNF-*α* and IL-6 were estimated by commercially available kits (BD Biosciences, USA).

#### 2.4.5. Histopathological Examination

The animals were sacrificed by overdose of xylazine 4.3 mg/kg (Indian immunological Ltd., India) and ketamine 25 mg/kg i.v. (Troikaa Pharmaceuticals Ltd., India). Eye ball was excised and fixed in 10% formalin solution. After 24 h, the tissues were washed thoroughly with repeated use of absolute alcohol. These cycles were repeated till the final sections cut with tissue. Later the eye ball was cut down in middle of optic nerve to the cornea. Then the sections of eye were kept in melted paraffin twice before drying. After they were dried, the block of paraffinized tissues was cut according to the size of tissue. Sections were deparaffinized in xylene and rehydrated through descending grades of alcohol. The sections were then stained with 10% hematoxylin for 3–5 minute and staining was intensified by placing in running water. Again sections were stained with 10% eosin for 2 minutes and quickly passed through ascending grade of alcohol. Finally they were treated with xylene followed by mounting and analyzing under OLYMPUS (trinocular-CX21FS1) microscope with 100X magnification.

#### 2.4.6. Analysis of H&E Stained Slide

The hematoxylin and eosin (H&E) stained slides were analyzed under photo microscope with 100x magnification setting. Eye boll section was observed for presence of retinal cell, blocking of trabecular meshwork (TM), epithelial cell matrix in mesh of TM, optic nerve head, and apoptosis.

### 2.5. Statistical Analysis

All the values were expressed as mean ± SEM. Statistics was applied using graph pad prism 6.0 (San Diego, CA, USA) for windows and SPSS statistical software (version 22). ANOVA was used to determine statistical significance between various groups. Difference was considered statistically significant when *p* < 0.05.

## 3. Results

### 3.1. Effects on IOP

Rise in IOP is the characteristic feature of glaucomatous state. Following changes were observed in IOP of acute and chronic model animals.

#### 3.1.1. Effect on IOP in Acute Model

**Figure 1:**
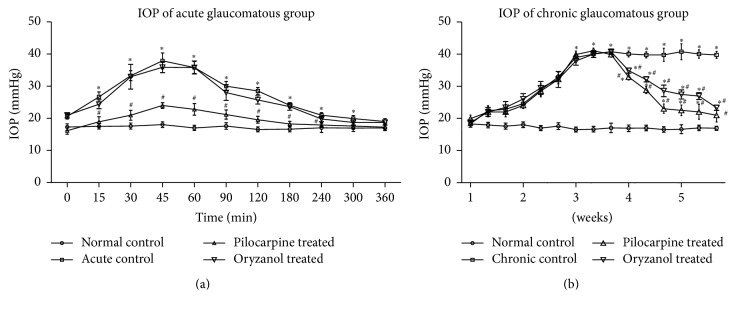
Effect of OZ on IOP. (a) Acute glaucomatous rabbits. (b) Chronic glaucomatous rabbits. Data are expressed as mean ± SEM. (*n* = 6). Values are statistically significant at ^*∗*^*p* < 0.05 versus* normal control*; ^#^*p* < 0.05 versus* chronic control*.

After treatment with 5% dextrose through marginal ear vein, in acute control group, significant (*p* < 0.05) elevation in IOP was observed compared to normal control group. IOP level was at peak at 45 minute and normalized by 4–6 hrs. In this model treatment with OZ did not reduce IOP in its respective treatment group, while pilocarpine treatment led to reducing the IOP significantly (*p* < 0.05) as compared to acute control group (Figure 1(a)).

#### 3.1.2. Effect on IOP in Chronic Model

After topical administration of 5% phenol for 3 weeks of induction period, IOP was found to be significantly (*p* < 0.05) increased to 38–42 mmHg as compared to normal control group (Figure 1(b)). In this model both pilocarpine and OZ treatment resulted in significant (*p* < 0.05) fall in IOP as compared to chronic control group.

### 3.2. Effects on Total Protein Level

**Figure 2:**
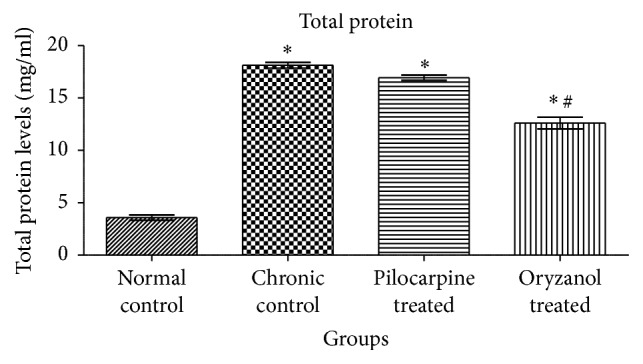
Effect of OZ on total protein concentration in AH of chronic glaucomatous rabbits. Data are expressed as mean ± SEM. (*n* = 6). Values are statistically significant at ^*∗*^*p* < 0.05 versus* normal control*; ^#^*p* < 0.05 versus* chronic control*.

Protein present in the AH has diagnostic significance as it is associated with pathogenesis of several ocular abnormalities. In current study significant (*p* < 0.05) elevation in total protein level was observed in chronic control group as compared to normal control. Significant (*p* < 0.05) reduction in total protein was observed in OZ treated group as compared to chronic control group (Figure 2).

### 3.3. Effect on Oxidative Stress Markers

**Figure 3:**
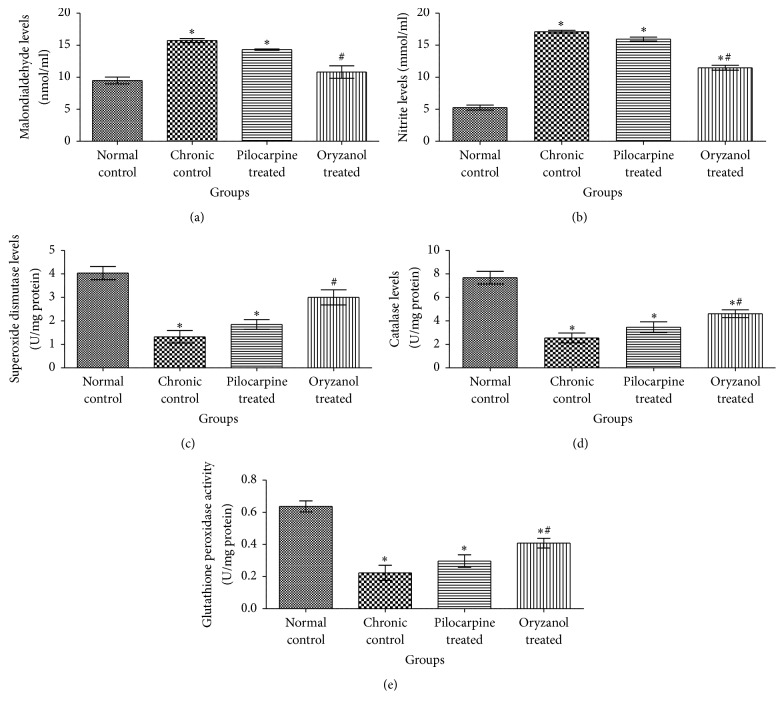
Effect of OZ on various oxidative stress markers. (a) Malondialdehyde (MDA) level, (b) nitrite (NO) level, (c) superoxide dismutase (SOD) level, (d) catalase (CAT) level, and (e) glutathione peroxidase GPx level. Data are expressed as mean ± SEM. (*n* = 6). Values are statistically significant at ^*∗*^*p* < 0.05 versus* normal control*; ^#^*p* < 0.05 versus* chronic control*.

Significant (*p* < 0.05) elevation of MDA and NO levels as well as significant (*p* < 0.05) reduction in SOD, CAT, and GPx levels was observed in chronic control group as compared to normal control group. Significant (*p* < 0.05) reduction in MDA and NO levels as well as significant (*p* < 0.05) elevation in SOD, CAT, and GPx levels was observed with OZ treatment when compared to chronic control animals (Figure 3).

### 3.4. Effects on Inflammatory Mediators

**Figure 4:**
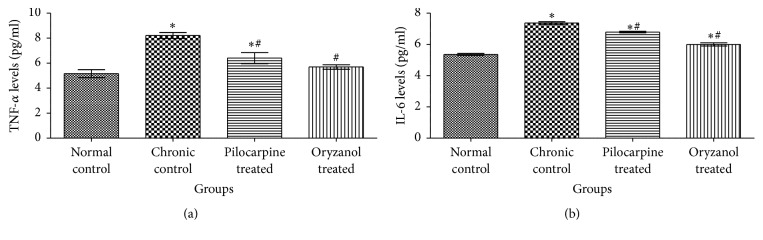
Effect of OZ on inflammatory mediators. (a) TNF-*α* level and (b) IL-6 level. Data are expressed as mean ± SEM. (*n* = 6). Values are statistically significant at ^*∗*^*p* < 0.05 versus* normal control*; ^#^*p* < 0.05 versus* chronic control*.

TNF-*α* and IL-6 are inflammatory markers. In our findings we observed significant (*p* < 0.05) elevation in TNF-*α* as well as IL-6 in chronic control animals as compared to normal control animals. OZ and pilocarpine treated groups showed significant (*p* < 0.05) reduction in inflammatory markers in their respective treatment groups as compared to chronic control group (Figure 4).

### 3.5. Histopathology

**Figure 5:**
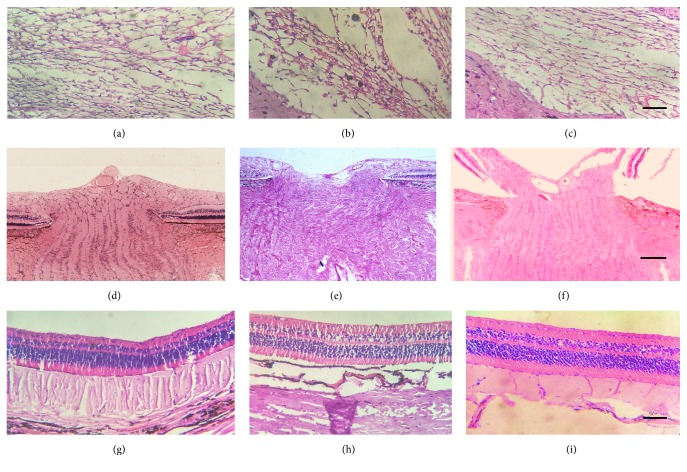
Histopathological sections of various tissues of rabbit's eye. Trabecular meshwork of (a) normal control, (b) chronic control, and (c) OZ treatment; optic nerve head (lamina cribrosa) of (d) normal control, (e) chronic control, and (f) OZ treatment; retina of (g) normal control, (h) chronic control, and (i) OZ treatment animals. (Magnification: 250x, scale: 150 *μ*m).

Histopathological examinations of eyes were performed to study trabecular meshwork, optic nerve head, and retinal cells by 100x power lens under inverted microscope using eosin and hematoxylin stain. Under histopathology examination, TM section of normal control animals revealed open passage for AH drainage. In contrast, eye sections of chronic control and OZ treated groups revealed irregular TM meshwork cells and aggregation of extracellular tissue fibers in pore of TM and canal of Schlemm (Figures 5(a), 5(b), and 5(c)). Extracelluar matrix aggregation was seen more on surface of TM in chronic control group as compared to normal control and OZ treated eyes. Canal of Schlemm and TM cellularity were found to be completely disturbed in both chronic control and OZ treated animals (Figures 5(b) and 5(c)). Further, the histopathological analysis of optic nerve head in normal control group showed no sign of cupping in lamina cribrosa (Figure 5(d)). Eye of chronic control animals exhibited complete change in the normal architecture of optic nerve head along with cupping and degradation of optic nerve head (Figure 5(e)). However, treatment with OZ exhibits less tissue degeneration and cupping on lamina cribrosa as compared to chronic control animal's eye (Figure 5(f)). H&E staining of the normal control animal's retina exhibited normal architecture (Figure 5(g)). Retina of both chronic control and OZ treated eyes revealed severe loss of RGCs in the temporal region, especially in mecula. The remaining structure and cellular components of the chronic control and OZ treated eyes appeared to stain within normal limits compared with staining pattern of normal control's retina. Chronic control group showed clear decrease in cellularity of the retina, mainly rod, cones, and polar cell degeneration; moreover outer layer of the retina was also disturbed completely as compared to normal control animal's eye (Figures 5(h) and 5(i)).

## 4. Discussion

In acute model of glaucoma transient elevation in IOP (38–42 mmHg) was observed, when 5% dextrose (15 ml/kg) was administered through marginal ear vein. Pretreatment with 1% OZ in acute model (OA) failed to produce any alteration in the raised IOP. In contrast 1% pilocarpine significantly (*p* < 0.05) prevented the acute rise in IOP as compared to control. The ineffectiveness of OZ on acute elevation of IOP indicates that OZ may not act through direct action on AH outflow pathways.

Experimentally induced chronic glaucoma mimics the moderate IOP elevation, a characteristic of human open angle glaucoma. It had already been stated that exposure of phenol to the murine skin can stimulate glutathione oxidation, vitamin- E level, and total antioxidant assets in skin epidermis. Phenol had also been stated to elevate the free radicle formation in RBCs in vitro [22]. After three weeks of induction period, significant elevation in the IOP was observed in all animals injected with 0.3 ml of 5% phenol in almond oil (*p* < 0.05) as compared to normal control animals. In our study after 2 weeks of 1% OZ treatment, significantly (*p* < 0.05) reduced IOP level was observed in chronic model of glaucoma. Pilocarpine treatment significantly (*p* < 0.05) reduced IOP as compared to disease control group. IOP, after treatment with pilocarpine, was comparable to that of normal control group. This activity is attributed to its cholinergic receptor agonistic activity. Pilocarpine decreases IOP by activating muscarinic receptors present on postsynaptic ciliary muscle and facilitating contraction of it and improves patency of fluid channels in the TM, therefore promoting trabecular outflow facility [23].

Normally anterior portion of eye and blood stream both remain separated by anatomic barrier formed by endothelial layer of blood vessels in the iris and nonpigmented cellular layer of ciliary epithelium so-called blood aqueous barrier (BAB). It regulates transference of protein by active transport. Glaucomatous condition leads to collapse of the barrier and promotes protein influx from serum into AH [14]. These elevated protein aggregates can block the regular AH drainage and elevate IOP. Subconjunctival injection of 5% phenol produces irritation and inflammatory reaction in eye, which damages the anterior epithelial and blood vessels and subsequently increases the protein concentration in anterior chamber. In current findings total protein in AH of glaucomatous animals was found to be approximately 18% higher than that in NC. 1% OZ treated animals showed significant (*p* < 0.05) reduction in total protein level in AH as compared to chronic control animals. Moreover, the change in the protein concentration matches the change in IOP for all studied groups. 1% pilocarpine treated group exhibited only mild reduction in protein level, which did not significantly differ from eyes of chronic control group.

Previous findings have reported role of oxidative stress markers in open angle glaucoma pathogenesis. ROS are substantially associated with oxidative damage of DNA in TM of glaucomatous victims. This uneven oxidant and antioxidant divergence promotes damage of TM, RGCs, and neuronal death. These changes follow chronic alterations in AH as well as vitreous humor (VH) [24]. In previous studies there was significantly high MDA and reduced SOD, CAT, and GPx levels were reported in rats with elevated IOP having ischemic retinal tissues [25]. In our findings chronic control group exhibited significant (*p* < 0.05) increase in the MDA level and relatively decreased SOD, CAT, and GSH as compared to normal control group. 1% OZ treated group exhibited significant (*p* < 0.05) reduction in MDA level as well as significantly (*p* < 0.05) improved SOD, CAT, and GPx level. However 1% pilocarpine treatment did not show significant reduction in MDA level and increment in SOD, CAT, and GPx levels when compared to chronic control animal.

Former studies anticipated manifestation of inducible nitric oxide synthase (iNOS) in optic nerve of human as well as experimental glaucomatous rat eyes. Reactive astrocytes produce excessive NO which is followed by degeneration of RGCs. The fetal role of NOS-2 and NO was recognized by treatment with iNOS specific inhibitor, amino guanidine, in rats with iatrogenically raised IOP and the findings validated the role of NO in pathogenesis of IOP prompted glaucomatous neuropathy of optic nerve and retinal ganglia [26]. In our findings nitrite level was significantly (*p* < 0.05) increased in chronic control group compared to normal control group. In previous study ethyl ester of ferulic acid exhibited significant reduction in iNOS activity [27]. Hence, it can be suggested that oryzanol may reduce nitrite level through modulation of iNOS expression and activity. Treatment with 1% OZ showed significant (*p* < 0.05) reduction in nitrite levels as compared to chronic control animals. According to reported studies OZ resists oxidative damage by its free radical scavenging activity which is due to presence of hydroxy and phenoxy group in its ferulic acid structure so that it can neutralize ROS by donating electron [28]. No significant change in nitrite level was observed in pilocarpine treated animals as compared to chronic control group. These findings suggest that OZ might prevent oxidative damage of RGCs more effectively than pilocarpine therapy.

TNF-*α* and IL-6 are hallmark of inflammation and are associated with inflammation induced damage of various tissues. Previously conducted studies reveal apparent upregulation of TNF-*α* facilitated signaling, which is associated with neurodegeneration in glaucomatous state [29]. In glaucomatous eye, glial cell mediated TNF-*α* production is augmented and this is followed by upregulation of death receptor on RGCs and series of events, that is, receptor-facilitated caspase activation, dysfunction of mitochondrial activity, and oxidative stress induced cell degeneration. Findings also support TNF-*α* upregulation in experimentally induced IOP in ocular hypertensive eyes [30]. Previous studies also support the elevation of IL-6 in AH of glaucomatous eye [31]. In present study significant (*p* < 0.05) elevation in TNF-*α* and IL-6 was found in chronic control animals as compared to normal control animals. In the line of previous reports, treatment with OZ and pilocarpine showed significant (*p* < 0.05) reduction in TNF-*α* and IL-6 [32].

The anterior section of the eye is organized with TM, a filter-like tissue which is made of a sequence of fenestrated beams by which AH flows for leaving the anterior chamber via canal of Schlemm. The TM is gathered to control the AH flow so as to create IOP. Dysregulated AH outflow leads to rise of IOP, and it is the principal risk factor for glaucomatous condition. The juxtacanalicular tissue (JCT) or cribriform region that is the part of TM and dwells adjacent to canal of Schlemm is associated with IOP regulation. Continuous remodeling of extracellular matrix (ECM) is more crucial for maintenance of AH flow channels through liberation of confined fragments and ECM associated debris from discharge passageways. The ECM concomitant with JCT prevents outflow hindrance by sequestration of small molecules and maintains IOP by regulating AH flow [33]. The neurolytic potency of phenol has been studied. It is reported to promote neurodegeneration by precipitation of protein, alteration of axonal myelin sheath structure, and damaging of fatty elements of nerve cells [34]. Further it has been reported that visual field defect in glaucomatous disorder follows both structural and functional impairment of optic nerve head as well as axonal damage. Rise in IOP is associated with physical change in optical nerve head which can be seen as cupping of optic disc and causes axonal compression of optic nerve at lamina cribrosa, axonal transport interruption, and hindrance of neurotrophin transportation followed by neuronal death. IOP is further related to structural and mechanical insults on astrocytes and their transformation into reactive form. These reactive astrocytes fail to provide structural support to optic nerve cells and produce matrix metalloproteinase that influences the matrix remodeling pattern [35, 36]. It is further strongly reported that interruption in ECM remodeling leads to augmented ECM material buildup in TM which resists the normal outflow of AH and raises IOP [37]. Our findings follow the reported cellular and structural modulation in glaucomatous state (Figure 5).

## 5. Conclusion

In support of given evidences it is clear that OZ treatment can prevent glaucoma by reducing elevated IOP and decrease optic nerve and retinal damage by controlling inflammatory mediators and scavenging free radicle.

## References

[1] Mozaffarieh M., Fraenkl S., Konieczka K., Flammer J. (2010). Targeted preventive measures and advanced approaches in personalised treatment of glaucoma neuropathy. *EPMA Journal*.

[2] Awoyesuku E. A., Fiebai B. (2011). Neuroprotection in glaucoma: a review. *The Nigerian Health Journal*.

[3] WHO Visual impairment and blindness. http://www.who.int/mediacentre/factsheets/fs282/en.

[4] Kuehn M. H., Fingert J. H., Kwon Y. H. (2005). Retinal ganglion cell death in glaucoma: mechanisms and neuroprotective strategies. *Ophthalmology Clinics of North America*.

[5] Pinal S., Vecino E., Westerhouse A. N. (2009). Current trends in glaucoma: what about neuroprotection?. *Eye Research Developments*.

[6] Patel M., Naik S. N. (2004). Gamma-oryzanol from rice bran oil—a review. *Journal of Scientific and Industrial Research*.

[7] Juliano C., Cossu M., Alamanni M. C., Piu L. (2005). Antioxidant activity of gamma-oryzanol: mechanism of action and its effect on oxidative stability of pharmaceutical oils. *International Journal of Pharmaceutics*.

[8] Islam M. S., Murata T., Fujisawa M. (2008). Anti-inflammatory effects of phytosteryl ferulates in colitis induced by dextran sulphate sodium in mice. *British Journal of Pharmacology*.

[9] Brambilla R., Dvoriantchikova G., Barakat D., Ivanov D., Bethea J. R., Shestopalov V. I. (2012). Transgenic inhibition of astroglial NF-*κ*B protects from optic nerve damage and retinal ganglion cell loss in experimental optic neuritis. *Journal of Neuroinflammation*.

[10] Ismail N., Ismail M., Imam M. U. (2014). Mechanistic basis for protection of differentiated SH-SY5Y cells by oryzanol-rich fraction against hydrogen peroxide-induced neurotoxicity. *BMC Complementary and Alternative Medicine*.

[11] Cheng C.-Y., Su S.-Y., Tang N.-Y., Ho T.-Y., Chiang S.-Y., Hsieh C.-L. (2008). Ferulic acid provides neuroprotection against oxidative stress-related apoptosis after cerebral ischemia/reperfusion injury by inhibiting ICAM-1 mRNA expression in rats. *Brain Research*.

[12] Reddy K. V. K., Maheswaraiah A., Naidu K. A. (2014). Rice bran oil and n-3 fatty acid-rich garden cress (*Lepidium sativum*) seed oil attenuate murine model of ulcerative colitis. *International Journal of Colorectal Disease*.

[13] Panchal S. S., Mehta A. A., Santani D. D. (2016). Effect of potassium channel openers in acute and chronic models of glaucoma. *Taiwan Journal of Ophthalmology*.

[14] Abdelkawi S., Ahmed M. (2012). Impact of elevated intraocular pressure and 0.15% brimonidine tartrate on aqueous humor and retina of experimental animal. *International Journal of Pharmacy and Pharmaceutical Sciences*.

[15] Gupta S. K., Kalaiselvan V., Agrawal S. S., Srivastava S., Saxena R. (2011). Prevention of endotoxin-induced uveitis in rabbits by Triphala an Ayurvedic formulation. *International Journal of Current Pharmaceutical Research*.

[16] Lowry O. H., Rosenbrough N. J., Farr A. L., Randall R. J. (1951). Protein measurement with the Folin-phenol reagent. *The Journal of Biological Chemistry*.

[17] Ohkawa H., Ohishi N., Yagi K. (1979). Assay for lipid peroxides in animal tissues by thiobarbituric acid reaction. *Analytical Biochemistry*.

[18] Tracey W. R., Tse J., Carter G. (1995). Lipopolysaccharide-induced changes in plasma nitrite and nitrate concentrations in rats and mice: pharmacological evaluation of nitric oxide synthase inhibitors. *Journal of Pharmacology and Experimental Therapeutics*.

[19] Mohandas M., Marshall J. J., Duggin G. G. (1984). Differential distribution of glutathione and glutathione related enzymes in rabbit kidney. *Cancer Research*.

[20] Kovačeva J., Pláteník J., Vejražka M. (2007). Differences in activities of antioxidant superoxide dismutase, glutathione peroxidase and prooxidant xanthine oxidoreductase/xanthine oxidase in the normal corneal epithelium of various mammals. *Physiological Research*.

[21] Hugo E. B. (1984). Oxidoreductase activity on groups other than CHOH-Catalase. *Methods in Enzymalogy*.

[22] El-Hossary G. G., El-Hohary A. A., El-Shazly A. H., Departments P. (2010). Topical instillation of aminoguanidine reducing intraocular pressure and improving visual evoked potential in rabbits with experimental glaucoma. *Research Journal of Medicine and Medical Sciences*.

[23] Sarchahi A. A., Gholipour M. A., Toghraie F. S. (2011). Effects of latanoprost and pilocarpine combination on the intraocular pressure and pupil size of normal rabbits. *Iranian Journal of Veterinary Research*.

[24] Oduntan O. A., Mashige K. P. (2011). A review of the role of oxidative stress in the pathogenesis of eye diseases. *South African Optometrist*.

[25] Tong N., Zhang Z., Gong Y., Yin L., Wu X. (2012). Diosmin protects rat retina. *Journal of Ocular Pharmacology and Therapeutics*.

[26] Kaufman P. L. (1999). Nitric-oxide synthase and neurodegeneration/neuroprotection. *Proceedings of the National Academy of Sciences of the United States of America*.

[27] Sultana R., Ravagna A., Mohmmad-Abdul H., Calabrese V., Butterfield D. A. (2005). Ferulic acid ethyl ester protects neurons against amyloid *β*-peptide(1–42)-induced oxidative stress and neurotoxicity: relationship to antioxidant activity. *Journal of Neurochemistry*.

[28] Srinivasan M., Sudheer A. R., Menon V. P. (2007). Ferulic acid: therapeutic potential through its antioxidant property. *Journal of Clinical Biochemistry and Nutrition*.

[29] Yang X., Luo C., Cai J. (2011). Neurodegenerative and inflammatory pathway components linked to TNF-*α*/TNFR1 signaling in the glaucomatous human retina. *Investigative Ophthalmology and Visual Science*.

[30] Tezel G. (2008). TNF-*α* signaling in glaucomatous neurodegeneration. *Progress in Brain Research*.

[31] Cvenkel B., Kopitar A. N., Ihan A. (2010). Inflammatory molecules in aqueous humour and on ocular surface and glaucoma surgery outcome. *Mediators of Inflammation*.

[32] Islam S., Nagasaka R., Ohara K. (2011). Biological abilities of rice bran-derived antioxidant phytochemicals for medical therapy. *Current Topics in Medicinal Chemistry*.

[33] Keller K. E., Acott T. S. (2013). The Juxtacanalicular Region of ocular trabecular meshwork: a tissue with a unique extracellular matrix and specialized function. *Journal of Ocular Biology*.

[34] Rowe D. S. (1989). *Neurolytic Techniques for Pain Management*.

[35] Osborne N. N., Melena J., Chidlow G., Wood J. P. M. (2001). A hypothesis to explain ganglion cell death caused by vascular insults at the optic nerve head: possible implication for the treatment of glaucoma. *British Journal of Ophthalmology*.

[36] Guo L., Moss S. E., Alexander R. A., Ali R. R., Fitzke F. W., Cordeiro M. F. (2005). Retinal ganglion cell apoptosis in glaucoma is related to intraocular pressure and IOP-induced effects on extracellular matrix. *Investigative Ophthalmology and Visual Science*.

[37] Tovar-Vidales T., Roque R., Clark A. F., Wordinger R. J. (2008). Tissue transglutaminase expression and activity in normal and glaucomatous human trabecular meshwork cells and tissues. *Investigative Ophthalmology and Visual Science*.

